# Supplementary material for: Control of motor landing and processivity by the CAP-Gly domain in the KIF13B tail
Source: Nat Commun. 2023 Aug 5;14:4715. doi: 10.1038/s41467-023-40425-4 (PMC10404244; doi:10.1038/s41467-023-40425-4)
Supplement: Supplementary file 1 — Supplementary Information [file 41467_2023_40425_MOESM1_ESM.pdf]

Control of Motor Landing and Processivity by the CAP-Gly Domain in the KIF13B Tail

Xiangyu Fan and Richard J. McKenney

Supplementary Information

Supplementary Methods

Plasmids and proteins

A DNA fragment encoding the mScarlet-2xStrepII tag was synthesized as a gBlock (Integrated DNA Technologies, Coralville, IA, USA). Coding sequences of human KIF13B^{K414A} was tagged with mScarlet-StrepII in C-terminal was cloned into pAceBac1 vector (Geneva Biotech) by Gibson Assembly. To generate KIF13B^{K414A}-mScarlet (I)-StrepII construct, a T to I mutation at position 74 in mScarlet was introduced by PCR-based mutagenesis. Full-length human KIF5B cDNA was purchased form Transomics (BC126281). To generate an insect cell expression construct for KIF5B^{CG} chimera, the coding sequences of human KIF5B (a.a. 1-912), KIF13B CAP-Gly domain (a.a 1695-1826) and sfGFP-StrepII were cloned into pAceBac1 vector (Geneva Biotech) by Gibson Assembly. All constructs were verified by Sanger sequencing.

The KIF5B^{CG} chimera was expressed in Sf9 cells and purified using the same methods described for KIF13B proteins. To generate the dual-color KIF13B motors, 200 ml of Sf9 cells were co-infected by P2 virus of HsKIF13B^{K414A}-sfGFP-StrepII and HsKIF13B^{K414A}-mScarlet-StrepII. Proteins were purified using the same methods described above.

Purified recombinant proteins KIF5B (1-912)-mScarlet-Strep, His-StrepII-sfGFP-HsKLC1 and HsKIF5B-2xPPS-mScarlet-StrepII are kind gifts from Kyoko Chiba and purified as described[1]. Recombinant protein p150-N-term (1-530) -mscarlet is a kind gift from Wenzhi Li. The protein was expressed and purified as described[2].

Protein sequences alignment

Protein sequences of KIF13B homologs were retrieved from diverse eukaryotic organisms using Uniprot Knowledgebase (UniprotKB), and subsequently aligned using Clustal WS with default settings of Jalview (Clustal W and Clustal X version 2.0)[3].

AlphaFold2 structure prediction

The structure of KIF13B^{CG} was performed using the ColabFold running on Google Colaboratory[4, 5], and generated structural models were prepared using ChimeraX[6].

Preparation of VASH1-SVBP treated microtubules

To prepare VASH1-SVBP treated detyrosinated microtubules, 10 nM of purified VASH1-SVBP complex were added into polymerized microtubules above (made with Alexa647-labelled tubulin) and freshly supplemented with 1 mM of PMSF and 1 mM of DTT. The mixture was incubated overnight at 37°C to remove the tyrosine at C-terminal of α-tubulin in microtubules. The reaction was terminated by the addition of 10 mM DTT, and the VASH1-SVBP complex was removed by centrifugation of the detyrosinated microtubules at 20, 000 x g over a 25% sucrose cushion. The microtubule pellet was resuspended as described above and the detyrosinated microtubules were used in TIRF assays as described above.

Analysis of the oligomerization state of KIF13B on microtubules by two-color single-molecule assays

Orthogonally labeled KIF13B^{K414A} (sfGFP or mScarlet tagged) were generated by co-infection of single SF9 cultures with baculoviruses encoding each motor variant. Cell cultures were infected with an equal volume of P2 virus supernatant and the infection and protein purification were carried out as described above. For single molecule assays, purified motors were introduced into the glass chamber pre-bounded with differentially labeled WT (tyrosinated) and CPA-treated (detyrosinated) microtubules. Movies were acquired as described above. Data were analyzed manually using ImageJ (Fiji), and statistical tests were performed in GraphPad Prism 9.

Analysis of the oligomerization state of KIF13B on microtubules by fluorescence intensity

Microtubules immobilized TIRF chambers were prepared as described above. Purified sfGFP-tagged protein was diluted to the indicated concentrations in the HP assay buffer with oxygen scavenging system in the presence of 2 mM ADP, then the diluted protein was introduced into the chamber. Movies were acquired using a Micromanager software-controlled Nikon TE microscope (1.49 numerical aperture, 100× objective) equipped with a TIRF illuminator and Andor iXon charge-coupled device electron-multiplying camera (405nm: 75% laser, 150 ms exposure, 488 nm: 50% laser, 150 ms exposure, time: 2 min, interval: no delay). Movies were subsequently analyzed by the image J (Fiji) plugin “SpotCounter”. Briefly, the first 200 frames in each movie were extracted and discarded to remove the signal from any aggregates and dead motors that are photobleached in those initial frame. Then, the fluorescence spots on selected microtubules or non-microtubule regions (KIF13B^{CG}) were analyzed by SpotCounter (Pre-filter: Gaussian, Box Size: 5, Noise Tolerance: 1100) [7, 8]. Statistical tests were performed in GraphPad Prism 9.

Supplementary Figures

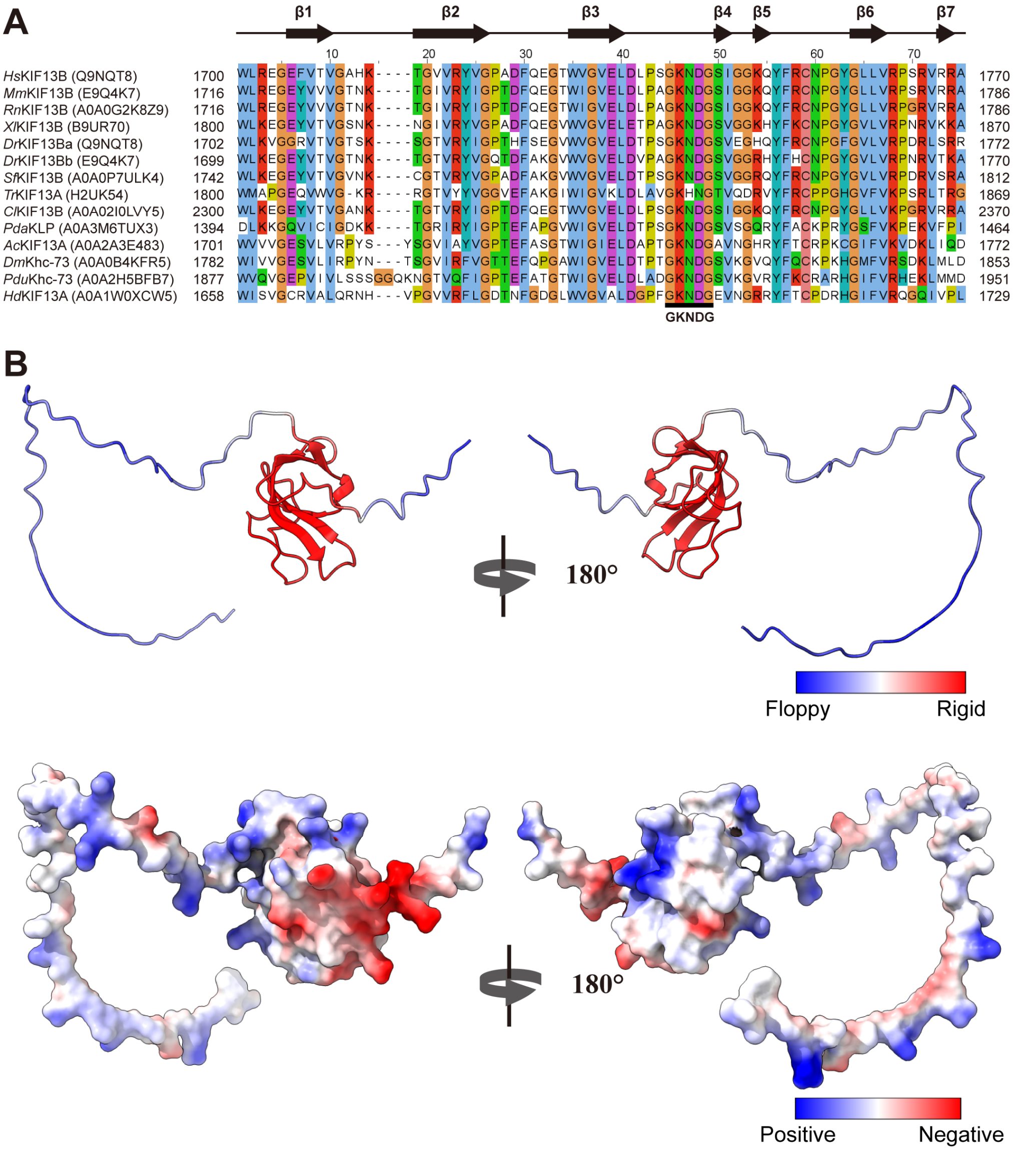

1685 QALASDSEEADEVPEWLREGEFVTVGAHKTGVVRYVGPADFQEGTWVGVELDLPSGKN 1742
1743 DGSIGGKQYFRCNPGYGLLVRPSRVRRATGPVRRRDGSIGGKQYFRCNPGYGLLVRPSR 1766
1767 VRRATGPVRRRSTGLRLGAPEARRSATLSGSATNLASLTAALAKADRSHKNPENRKSWAS 1826

Supplementary Figure 1. The CAP-Gly domain of KIF13B is evolutionary conserved. A Alignment of CAP-Gly domain protein sequences in KIF13B homologues. The predicted secondary structure is shown above, arrows and lines representing β- strands and random coils respectively. The core GKNDG motif within CAP-Gly domain was underlined by black[9]. The abbreviations of species names and protein names, as well as UniProtKB accession numbers (in brackets) are indicated in the left side before the protein sequences. Numbers show the positions of residues in corresponding protein sequences. Species names and abbreviations: *Homo sapiens (Hs), Mus musculus (Mm), Rattus norvegicus (Rn), Xenopus laevis (Xl), Danio rerio (Dr), Scleropages formosus (Sf), Takifugu rubripes (Tr), Columba livia (Cl), Pocillopora damicornis (Pda), Apis cerana (Ac), Drosophila melanogaster (Dm), Platynereis dumerilii (Pdu), Hypsibius dujardini (Hd)*. **B** The AlphaFold2 predicted structure of human KIF13B^{CG} truncation (a.a. 1685-1826). Top, Residues are colored by b-factor (theoretical), in which mobile and rigid residues are colored blue and red respectively. Middle, Residues are colored by electrostatic potential, in which positively charged and negatively charged residues are colored blue and red respectively. Bottom, protein sequence of KIF13B^{CG} truncation, the CAP-Gly domain is underlined, positively charged residues in the C-terminal of KIF13B are colored blue. Numbers show the positions of residues in corresponding human protein sequence.

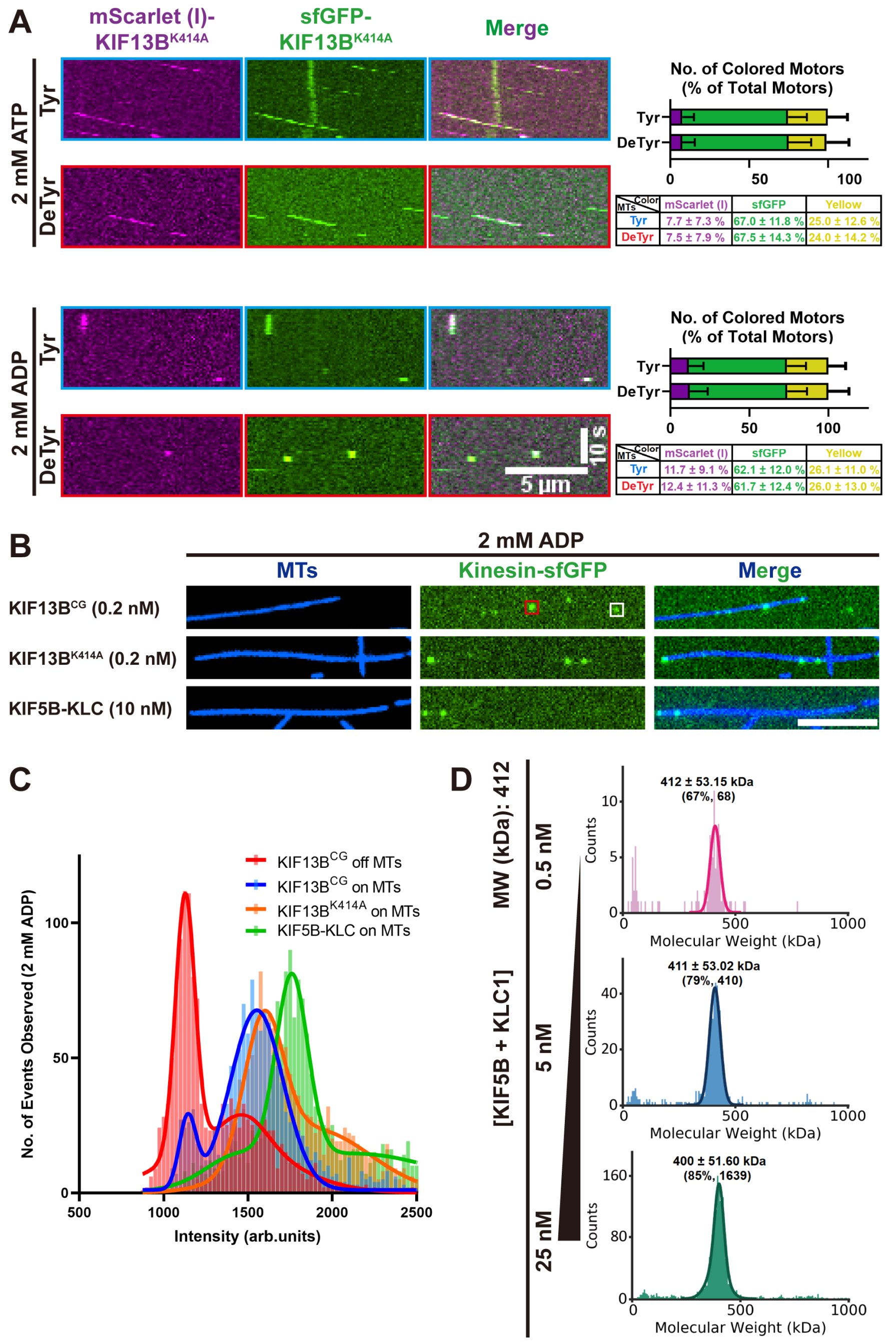

Supplementary Figure 2. Characterization of the oligomeric state of KIF13B^{K414A} and KIF13B^{CG} on microtubules. A Left, kymographs showing the KIF13B^{K414A}-mScarlet (I) and KIF13B^{K414A}-sfGFP on both types of microtubules. Scale bars: 10 s and 5 µm. Right, Quantification of the numbers of different colored motors on microtubules relative to total number of motors. The molar ratio of KIF13B^{K414A} is ~ 1:5 (mScarlet (I): sfGFP) by absorbance at each fluorophore wavelength. Given the molar ratio, the expected observation of each color motor is ~ 8% mScarlet (I), 17% mScarlet (I)/sfGFP, and ~ 75% sfGFP, consistent with our measurements and indicative that nearly all the motors on microtubules are dimers. The observed percentage of each color motor are shown in the table. Microtubules were quantified for each condition from two independent experiments. For quantification, n = 40 microtubules of each type (tyrosinated and detyrosinated). **B** TIRF images of microtubules (blue, left panel) and sfGFP fused KIF13B proteins or KIF5B-KLC complex (green, middle panel) in the presence of 2 mM ADP. Examples of proteins bound to microtubules are highlighted in the red box, and off microtubules by the white box (top middle panel). Scale bar: 5 µm. **C** Quantification of the oligomeric state of KIF13B^{K414A} on microtubules in the presence of 2 mM ADP. The fluorescence intensities of sfGFP fused proteins on MTs was compared with KIF13B^{CG} off MTs (monomer) and KIF5B-KLC (kinesin light chain) on MTs (dimer) respectively. Histograms of the sfGFP fluorescence intensity were plotted for each population of corresponding recombinant proteins and fit to multiple Gaussian distributions (N = 2 replicates). KIF13B^{CG} off MTs: n= 1326. KIF13B^{CG} on MTs: n= 1332. KIF13B^{K414A} off MTs: n= 1389. KIF13BK^{414A} on MTs: n= 1377. KIF5B-KLC on MTs: n= 1561. **D** Characterization of the oligomeric state of KIF5B-KLC complex by mass photometry. Histograms show the particle counts of KIF5B-KLC complex at the indicated molecular mass. Gaussian fits to the peaks are shown. Theoretical molecular weight of KIF5B-KLC is shown for comparison. For each concentration, the measurement was performed two times.n= 111, 532 and 1953 molecules (0.5 nM, 5 nM and 25 nM).

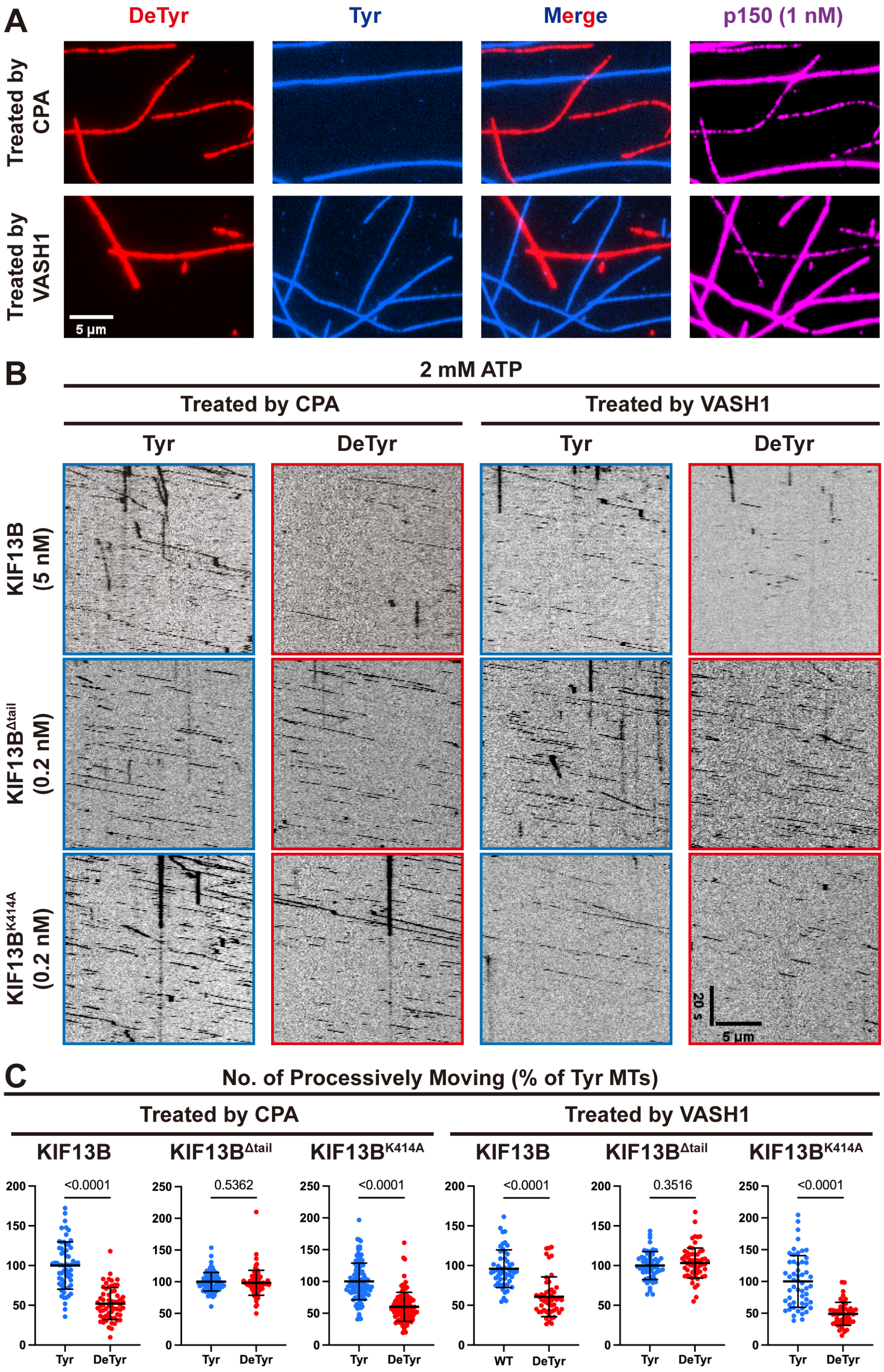

Supplementary Figure 3. Quantification of recombinant KIF13B motors on detyrosinated microtubules treated by CPA or VASH1-SVBP. A CPA-treated (top panel, red) or VASH1-SVBP treated (bottom panel, red) microtubules were mixed with untreated microtubules (blue) and introduced into same chamber. As a control, 1 nM of p150 N-terminal truncation (1-530) (magenta) was added and incubated 10 min at room temperature and subsequently visualized by TIRF microscopy. Scale bar: 5 µm. **B** Representative kymographs showing movements of KIF13B motors on tyrosinated (top panel) or detyrosinated microtubules (bottom panel). Left two panels, detyrosinated microtubule treated by CPA. Right two panels, detyrosinated microtubule treated by VASH1-SVBP. Scale bars: 20s and 5 µm. **C** Quantification of the number of processive motors of KIF13B motors per µm MT per second on tyrosinated or detyrosinated microtubules relative to tyrosinated microtubules in the same chamber. Top panels, detyrosinated microtubule treated by CPA (data were re-plotted from figure 3C for comparison). Bottom panels, detyrosinated microtubule treated by VASH1-SVBP. For the quantification of microtubules treated by VASH1-SVBP, microtubules were quantified for each condition from two independent experiments. KIF13B: n = 52 (both tyrosinated and detyrosinated microtubules). KIF13B$^{\Delta tail}$: n = 56 (both tyrosinated and detyrosinated microtubules). KIF13B^{K414A}: n = 54, 51 (tyrosinated and detyrosinated microtubules respectively). Mean ± SD are shown. *P* values are calculated from an unpaired, two-tailed *t*-test.

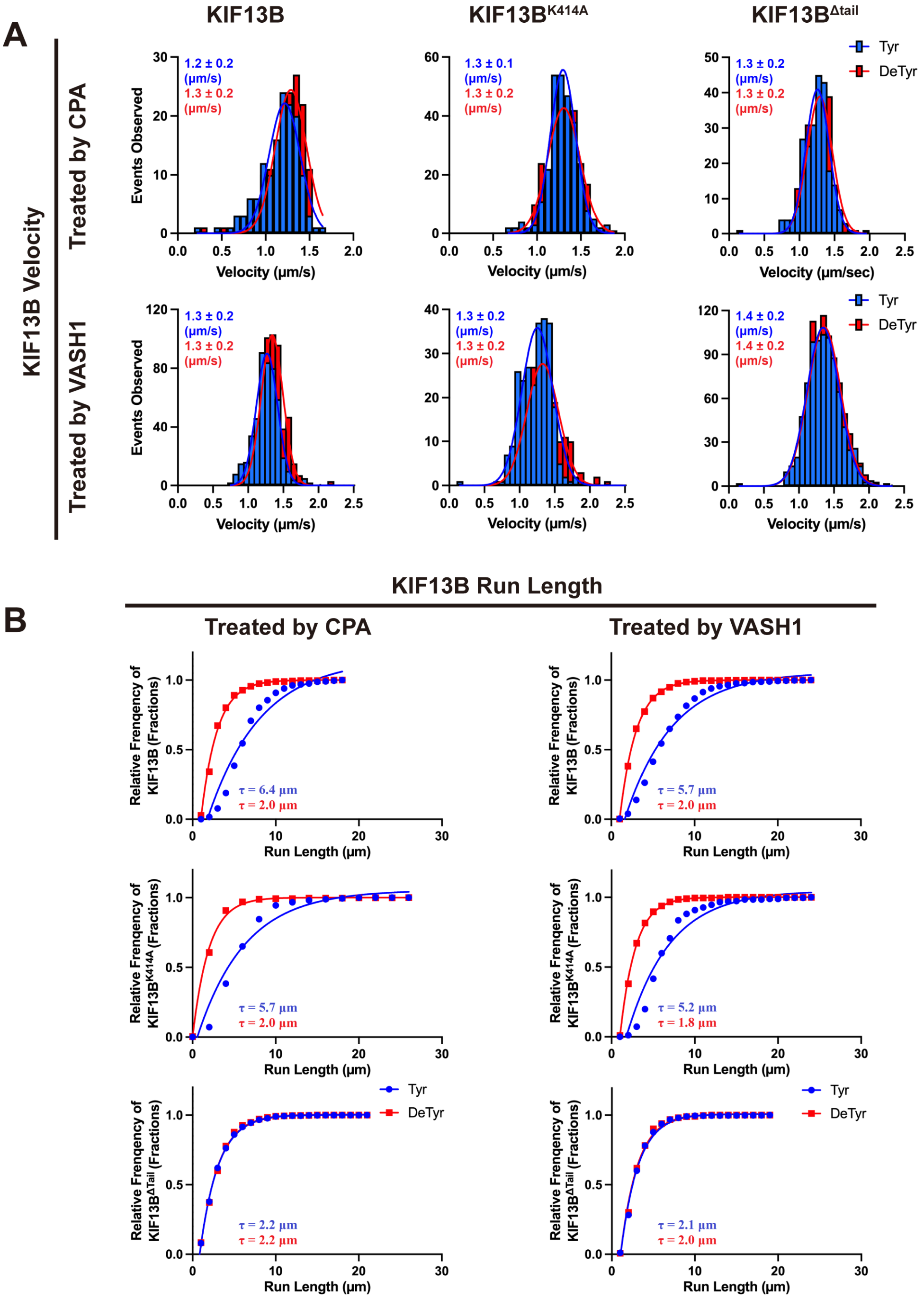

Supplementary Figure 4. Comparison the motile parameters of recombinant KIF13B motors on detyrosinated microtubules treated by CPA or VASH1-SVBP. A Quantification of the velocities of KIF13B motors on tyrosinated and detyrosinated microtubules. Histograms of the velocities were plotted for each population of motors and fit to a single Gaussian. The peak represents the average velocity of KIF13B and corresponding mutants. Top panels, detyrosinated microtubule treated by CPA (data were re-plotted from figure 3D for comparison). Bottom panels, detyrosinated microtubule treated by VASH1-SVBP. For the quantification of motors on VASH1-SVBP treated microtubules, events were quantified for each condition from two independent experiments. The mean ± SD for wild-type and each mutant of KIF13B are indicated. KIF13B: n = 476, 564 (on tyrosinated and detyrosinated microtubules respectively). KIF13B$^{\Delta tail}$: n = 838, 854. KIF13B^{K414A}: n = 262, 213. **B** Quantification of the run length of KIF13B motors on tyrosinated and detyrosinated microtubules. Cumulative frequency of the run lengths is plotted for each population of motors and fit to a one-phase exponential decay function. Left panels, detyrosinated microtubule treated by CPA (data were re-plotted from figure 3E for comparison). Right panels, detyrosinated microtubule treated by VASH1. For the quantification of motors on VASH1-treated microtubules, events were quantified for each condition from two independent experiments. The characteristic run lengths derived from the fits (τ) are indicated. KIF13B: n = 476, 564 and R^2 = 0.978, 0.9995 (on tyrosinated and detyrosinated microtubules respectively). KIF13B$^{\Delta tail}$: n = 838, 854 and R^2 = 0.992, 0.993. KIF13B^{K414A}: n = 262, 213 and R^2 = 0.956, 0.998.

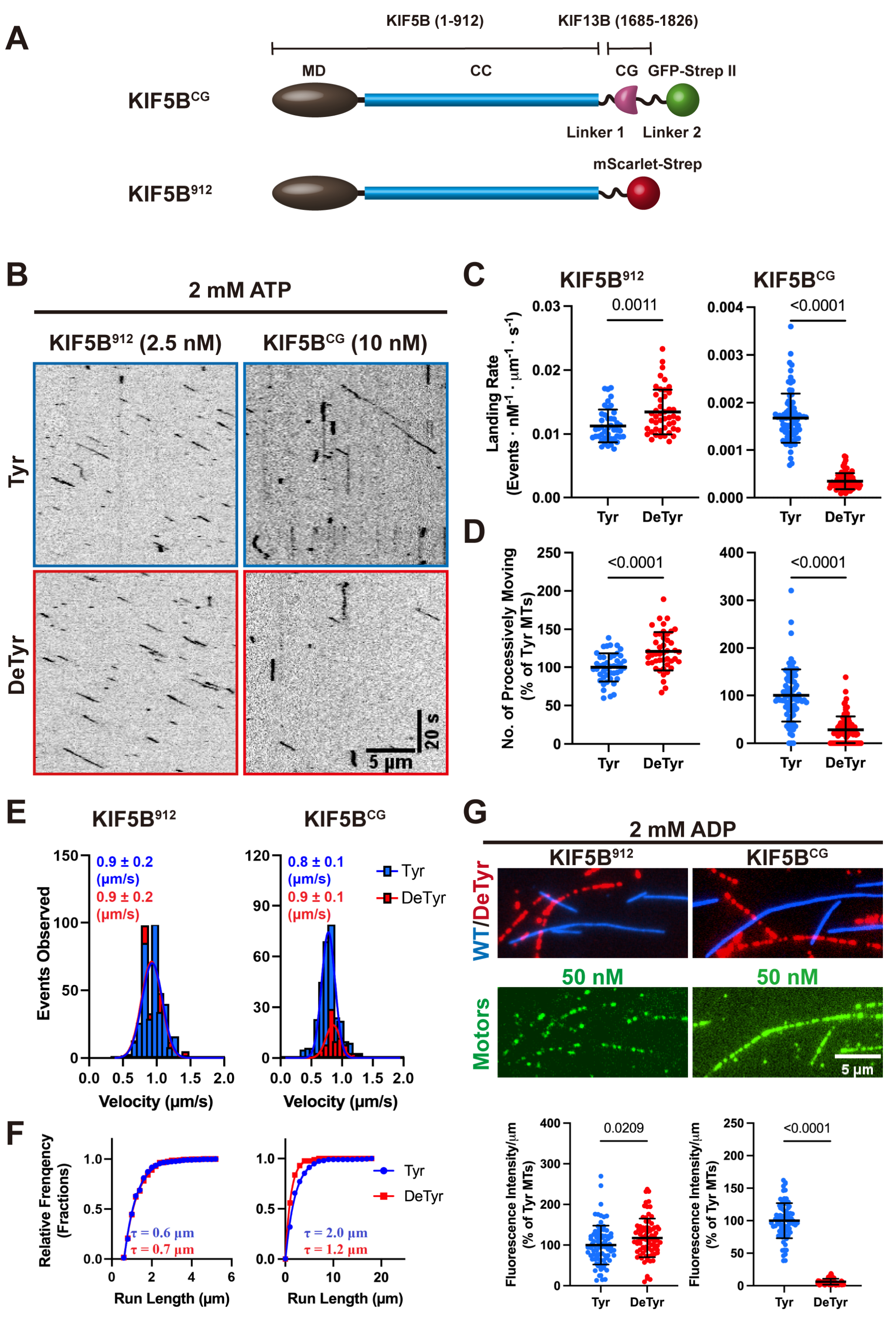

Supplementary Figure 5. Exogenous CAP-Gly domain affects kinesin-1 motility. **A** Schematic of KIF5B constructs. MD: motor domain, CC: coiled-coil, CG: CAP-Gly domain. **B** Kymographs showing movements of KIF5B^{912} and KIF5B^{CG} on tyrosinated (top) or CPA treated detyrosinated microtubules (bottom). Scale bars: 20 s and 5 µm. **C** Landing rates of KIF5B^{912} motor and chimeric motor on tyrosinated and detyrosinated microtubules. For both C and D, microtubules were quantified from two independent experiments. KIF5B^{912}: n = 45, KIF5B^{CG}: n = 80 (both tyrosinated and detyrosinated microtubules). Mean ± SD are shown. *P* values are calculated from an unpaired, two-tailed *t*-test. **D** Quantification of processive motors of KIF5B^{912} and KIF5B^{CG} on tyrosinated or detyrosinated microtubules relative to tyrosinated microtubules in the same chamber. Mean ± SD are shown. *P* values are calculated from an unpaired, two-tailed *t*-test. **E** Quantification of motor velocities on tyrosinated and detyrosinated microtubules. Histograms of the velocities were plotted for each population of motors and fit to a single Gaussian. Events quantified from two independent experiments. KIF5B^{912}: n = 360, 358 (on tyrosinated and detyrosinated microtubules), KIF5B^{CG}: n = 306, 86. The mean velocity ± SD for KIF5B^{912} and KIF5B^{CG} are indicated. **F** Cumulative frequency of the run lengths and fits to a one-phase exponential decay function. Events were quantified for each condition from two independent experiments. The characteristic run lengths (τ) are indicated. KIF5B^{912} n = 360, 358 and R^2 = 0.996, 0.996 (on tyrosinated and detyrosinated microtubules respectively), KIF5B^{CG}: n = 306, 86 and R^2 = 0.996, 0.999. **G** Top panel, TIRF images of motors (green, middle panel) bound to either tyrosinated (blue, top) or detyrosinated microtubules (red, top). Bottom, quantification of mean fluorescence intensity (arbitrary units) per µm microtubules for motors bound to tyrosinated or detyrosinated microtubules relative to tyrosinated microtubules in the same chamber. The mScarlet (I) signal of KF5B^{912} is displayed in green for consistency. Microtubules were quantified from two independent experiments. n = 80 for all conditions. Scale bar: 5 µm. Mean ± SD are shown. *P* values are calculated from an unpaired, two-tailed *t*-test.

Supplementary References

1. Chiba, K., Ori-McKenney, K. M., Niwa, S. & McKenney, R. J. Synergistic autoinhibition and activation mechanisms control kinesin-1 motor activity. *Cell Rep* **39**, 110900 (2022).
2. McKenney, R. J., Huynh, W., Vale, R. D. & Sirajuddin, M. Tyrosination of alpha-tubulin controls the initiation of processive dynein-dynactin motility. *EMBO J.* **35**, 1175-1185 (2016).
3. Larkin, M. A. *et al.* Clustal W and Clustal X version 2.0. *Bioinformatics* **23**, 2947-2948 (2007).
4. Jumper, J. *et al.* Highly accurate protein structure prediction with AlphaFold. *Nature* **596**, 583-589 (2021).
5. Mirdita, M. *et al.* ColabFold: making protein folding accessible to all. *Nat Methods* **19**, 679-682 (2022).
6. Pettersen, E. F. *et al.* UCSF ChimeraX: Structure visualization for researchers, educators, and developers. *Protein Sci* **30**, 70-82 (2021).
7. Edelstein, A., Amodaj, N., Hoover, K., Vale, R. & Stuurman, N. Computer control of microscopes using microManager. *Curr Protoc Mol Biol* **Chapter 14**, Unit14 20 (2010).
8. Edelstein, A. D. *et al.* Advanced methods of microscope control using muManager software. *J Biol Methods* **1**, (2014).
9. Weisbrich, A. *et al.* Structure-function relationship of CAP-Gly domains. *Nat. Struct. Mol. Biol.* **14**, 959-967 (2007).
