# Supplementary material for: Control of motor landing and processivity by the CAP-Gly domain in the KIF13B tail
Source: Nat Commun. 2023 Aug 5;14:4715. doi: 10.1038/s41467-023-40425-4 (PMC10404244; doi:10.1038/s41467-023-40425-4)
Supplement: Supplementary file 3 — Reporting Summary [file 41467_2023_40425_MOESM3_ESM.pdf]

nature portfolio

Corresponding author(s): Richard J. McKenney

Last updated by author(s): Jul 17, 2023

Reporting Summary

Nature Portfolio wishes to improve the reproducibility of the work that we publish. This form provides structure for consistency and transparency in reporting. For further information on Nature Portfolio policies, see our Editorial Policies and the Editorial Policy Checklist.

Statistics

For all statistical analyses, confirm that the following items are present in the figure legend, table legend, main text, or Methods section.

n/a	Confirmed	
☐	☒	The exact sample size (*n*) for each experimental group/condition, given as a discrete number and unit of measurement
☐	☒	A statement on whether measurements were taken from distinct samples or whether the same sample was measured repeatedly
☐	☒	The statistical test(s) used AND whether they are one- or two-sided<br>*Only common tests should be described solely by name; describe more complex techniques in the Methods section.*
☒	☐	A description of all covariates tested
☒	☐	A description of any assumptions or corrections, such as tests of normality and adjustment for multiple comparisons
☐	☒	A full description of the statistical parameters including central tendency (e.g. means) or other basic estimates (e.g. regression coefficient) AND variation (e.g. standard deviation) or associated estimates of uncertainty (e.g. confidence intervals)
☐	☒	For null hypothesis testing, the test statistic (e.g. *F*, *t*, *r*) with confidence intervals, effect sizes, degrees of freedom and *P* value noted<br>*Give P values as exact values whenever suitable.*
☒	☐	For Bayesian analysis, information on the choice of priors and Markov chain Monte Carlo settings
☒	☐	For hierarchical and complex designs, identification of the appropriate level for tests and full reporting of outcomes
☒	☐	Estimates of effect sizes (e.g. Cohen's *d*, Pearson's *r*), indicating how they were calculated

Our web collection on statistics for biologists contains articles on many of the points above.

Software and code

Policy information about availability of computer code

Data collection	Single molecule mass photometry data were collected at an acquisition rate of 1 kHz for 100s by Acquire MP (Refeyn).<br>Single molecule TIRF images were acquired using a Nikon NIS-Elements software-controlled (AR, Version 5.20.2) controlled TI-2 microscope (1.49 numerical aperture, 100× objective) equipped with a TIRF illuminator and Andor iXon charge-coupled device electron multiplying camera.<br>For the protein sequences alignment in supplementary materials, protein sequences of KIF13B homologs were retrieved from diverse eukaryotic organisms using Uniprot Knowledge base (UniprotKB).
Data analysis	Single molecule mass photometry data were analyzed by Discover MP (Refeyn).<br>For other assays, data were analyzed manually using Image J (FIJI Version 1.53t) and statistical tests were performed in GraphPad Prism 9.2.0.<br>For the protein sequences alignment in supplementary materials, protein sequences of KIF13B homologs were aligned using Clustal WS with default settings of Jalview Version 2.11.2.7 (Clustal W and Clustal X version 2.0).<br>For CAP-Gly domain structure prediction in supplementary materials, protein sequences of KIF13B CAP-Gly domain were predicted using the ColabFold Version 1.5.2 running on Google Colaboratory, and generated structural models were prepared using ChimeraX Version 1.5.

For manuscripts utilizing custom algorithms or software that are central to the research but not yet described in published literature, software must be made available to editors and reviewers. We strongly encourage code deposition in a community repository (e.g. GitHub). See the Nature Portfolio guidelines for submitting code & software for further information.

Data

Policy information about availability of data

All manuscripts must include a data availability statement. This statement should provide the following information, where applicable:

- Accession codes, unique identifiers, or web links for publicly available datasets
- A description of any restrictions on data availability
- For clinical datasets or third party data, please ensure that the statement adheres to our policy

All primary data used in this study are available within in the figures and from the authors upon reasonable request. Source data for all figures are included in the Source Data file.

Human research participants

Policy information about studies involving human research participants and Sex and Gender in Research.

Reporting on sex and gender	No Human participant involved in this syudy.
Population characteristics	N/A
Recruitment	N/A
Ethics oversight	N/A

Note that full information on the approval of the study protocol must also be provided in the manuscript.

Field-specific reporting

Please select the one below that is the best fit for your research. If you are not sure, read the appropriate sections before making your selection.

☒ Life sciences ☐ Behavioural & social sciences ☐ Ecological, evolutionary & environmental sciences

For a reference copy of the document with all sections, see nature.com/documents/nr-reporting-summary-flat.pdf

Life sciences study design

All studies must disclose on these points even when the disclosure is negative.

Sample size	Sample sizes were not preselected. Sample sizes for experiments were chosen to be as large as possible whilst producing datasets of a practical size for use in subsequent analysis with meaningful statistical relevance.
Data exclusions	For the single molecule TIRF assays, all events dwell time shorter than 300 ms were excluded to remove any non-specific binding during the quantification.
Replication	For all experiments, at least two independent experiments were reproduced across multiple protein preparations providing measurements of many biologically distinct samples (individual molecules or microtubules) for statistical analysis.
Randomization	All biological protein samples and reagents were aliquoted into small volumes and randomly utilized in each assay replicate. For imaging experiments, random fields of view were selected during imaging and molecular events were analyzed from random microtubules in the field of view.
Blinding	It is standard in the field of biochemistry that experimenter blinding is not relevant. The experiments were inherently blinded since it is impossible to select specific molecules from stock solutions when pipetting.

Reporting for specific materials, systems and methods

We require information from authors about some types of materials, experimental systems and methods used in many studies. Here, indicate whether each material, system or method listed is relevant to your study. If you are not sure if a list item applies to your research, read the appropriate section before selecting a response.

Materials & experimental systems

n/a	Involved in the study
☒	☐ Antibodies
☐	☒ Eukaryotic cell lines
☒	☐ Palaeontology and archaeology
☒	☐ Animals and other organisms
☒	☐ Clinical data
☒	☐ Dual use research of concern

Methods

n/a	Involved in the study
☒	☐ ChIP-seq
☒	☐ Flow cytometry
☒	☐ MRI-based neuroimaging

Eukaryotic cell lines

Policy information about cell lines and Sex and Gender in Research

Cell line source(s)	Insect SF9 cells(Gibco)
Authentication	Cell lines were used for protein expression and not authenticated for this study.
Mycoplasma contamination	Insect SF9 cells used in this study were not tested for mycoplasma contamination.
Commonly misidentified lines (See ICLAC register)	None
